# Primary Signet-Ring Cell Adenocarcinoma of the Urinary Bladder: A Report of a Rare Case

**DOI:** 10.7759/cureus.110980

**Published:** 2026-06-16

**Authors:** Gumie Bui, Isha Jaiswal, Lalit Kumar, Cijo Sanal, Gogul V Priean, Lahunshisha Kharbuli

**Affiliations:** 1 Radiation Oncology, Lakhimpur Cancer Centre, North Lakhimpur, IND; 2 Radiotherapy and Radiation Medicine, Institute of Medical Sciences, Banaras Hindu University, Varanasi, IND; 3 Urology, Institute of Medical Sciences, Banaras Hindu University, Varanasi, IND; 4 Radiation Oncology, Deccan College of Medical Sciences, Hyderabad, IND

**Keywords:** adenocarcinoma, bladder cancer, chemotherapy, radical cystectomy, radiotherapy, signet ring cell carcinoma

## Abstract

Primary signet-ring cell carcinoma (PSRCC) of the urinary bladder is a rare histological variant and is often diagnosed at an advanced stage. Here we are reporting a case of a 48-year-old male who presented to us with painless hematuria, which was associated with increased frequency of urination and a sensation of incomplete bladder emptying. On diagnostic evaluation, it was confirmed as PSRCC of the urinary bladder. The patient underwent radical cystectomy in view of recurrent disease and subsequently received radiation therapy with concurrent cisplatin. Follow-up after completion of treatment revealed the development of skeletal and penile metastases. The patient is currently 12 months post chemoradiation and was started on chemotherapy. This case highlights the aggressive clinical course of this rare malignancy and emphasizes the multimodality management and close follow-up.

## Introduction

Primary signet-ring cell carcinoma (PSRCC) of the urinary bladder is an extremely rare and aggressive subtype of bladder adenocarcinoma, comprising less than 1% of all primary bladder neoplasms [1]. The most common type is urothelial carcinoma, which occurs in 90% of all cases [2]. The other histological variants of bladder tumor include squamous cell carcinoma, adenocarcinoma, small cell carcinoma, micropapillary, sarcomatoid, plasmacytoid, and nested variants, which often present as aggressive tumors [3]. PRSCC of the bladder was first described by Otto Saphir in 1955, and to date, its incidence is very low [4].

PSRCC most commonly occur in the stomach and colon, usually presenting at advanced stages following asymptomatic progression [4] with a more aggressive biologic phenotype [5] and thereby resulting in a poorer prognosis for such patients. Extensive evaluation for the diagnosis of bladder PSRCC is required to rule out metastatic adenocarcinomas [6] and determine the primary origin of the cancer. Currently, surgery such as transurethral resection of bladder tumor (TURBT), partial cystectomy, and radical cystectomy remains the primary approach [7]. Radical cystectomy is the preferred surgical method. Given the aggressive clinical course of bladder SRCCs, management frequently involves radical cystectomy combined with chemotherapy and/or radiotherapy (RT). However, due to the rarity of the disease, there are currently no established guidelines or standardized recommendations regarding the role of adjuvant treatment [8]. This report describes a rare case of PRSCC of the urinary bladder managed with a multidisciplinary, multimodality treatment strategy.

## Case presentation

A 48-year-old male initially presented to the urology outpatient department with complaints of episodic hematuria, which was painless and associated with increased urinary frequency, incomplete bladder emptying, and the presence of amorphous clots for a two-month duration. The medical and familial history was unremarkable. He gave a history of tobacco chewing and bidi smoking for the last 10 years. Abdominal and systemic examination were normal. Local examination showed a mild left-sided hydrocele. Digital rectal examination revealed grade 2 prostatomegaly, which was firm in consistency.

The abdominal ultrasound revealed left kidney moderate hydroureteronephrosis with a 5 mm thickened and irregular urinary bladder wall. Magnetic resonance imaging (MRI) of the pelvis showed a 5.8 x 4.1 x 3.5 cm ill-defined endo-exophytic enhancing proliferative lesion with lobulated margins involving the bladder wall with involvement of the prostate and bilateral seminal vesicles. His routine laboratory workup was within normal limits. The patient initially underwent a TURBT and transurethral resection of the prostate (TURP), with cystoscopic findings revealing a 5 x 4 cm solid-looking broad-based growth over the left lateral wall extending up to the bladder neck and prostatic urethra. A deep biopsy was taken and sent for histopathological examination (HPE), which was suggestive of high-grade urothelial carcinoma. Due to incomplete clearance in the initial procedure, a repeat TURBT was performed with cystoscopy findings revealing a 3 x 2 cm papillary growth over the left lateral wall extending up to the bladder neck and prostatic urethra, and this time the HPE revealed signet-ring cell adenocarcinoma with lamina propria and muscularis propria invasion.

With no relief in symptoms, radical cystectomy followed by adjuvant therapy was planned. A metastatic workup with contrast-enhanced computed tomography (CECT) of the thorax and whole abdomen was performed, which showed no metastases elsewhere in the body. Cystoscopy showed residual disease of size 1 x 1 cm over the prostate fossa and growth of size 0.5 x 1 cm present on the right lateral bladder wall. The patient then underwent radical cystectomy with ileal conduit and bilateral extended pelvic lymph node dissection. Intraoperative findings showed a thickened bladder wall with a palpable hard mass at the level of the bladder neck and an enlarged left-sided lymph node.

**Figure 1:**
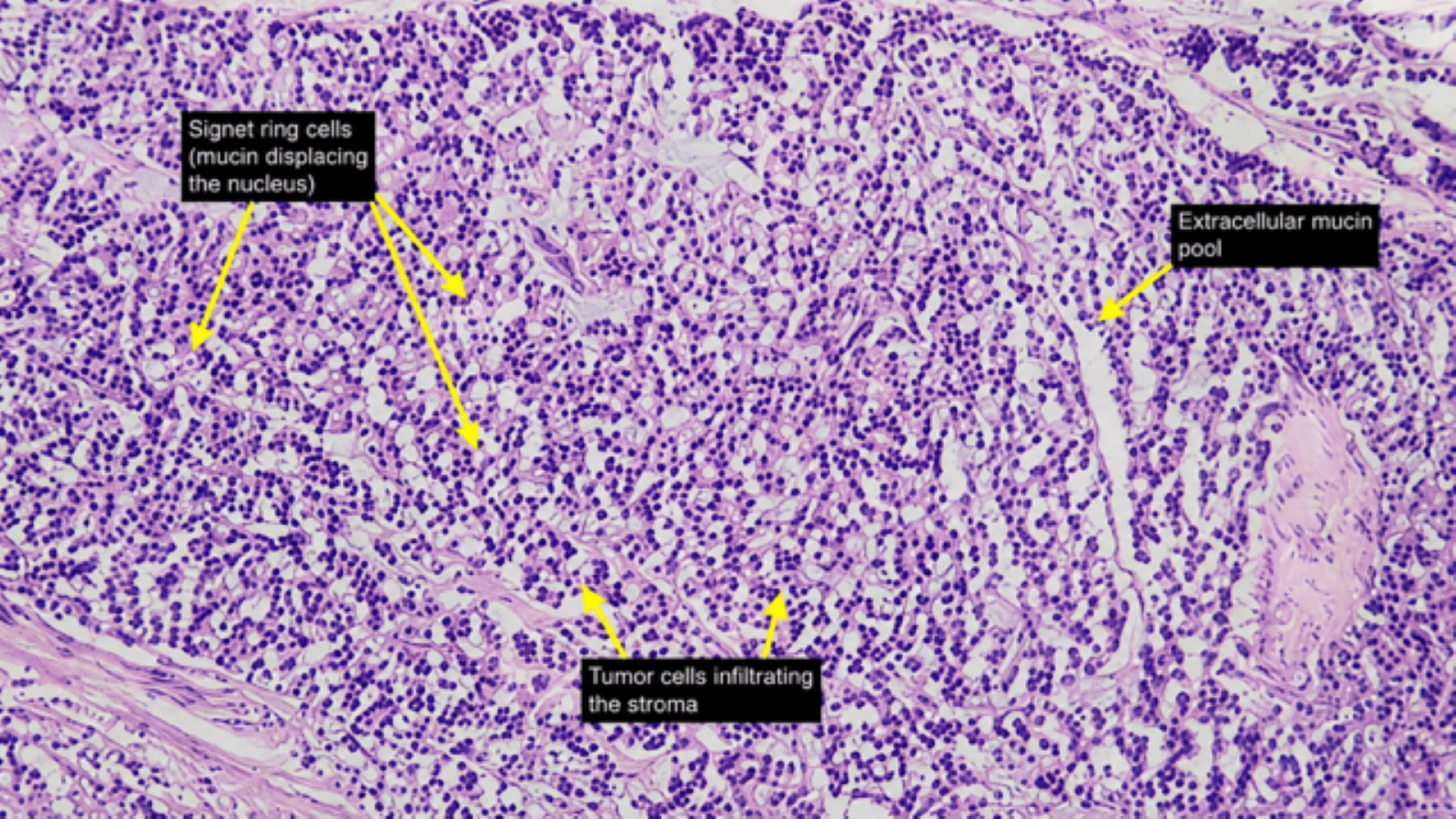
Histopathological image of signet-ring cell carcinoma of bladder

Postoperative HPE revealed a poorly differentiated signet-ring cell adenocarcinoma with invasion of muscle, perivesical soft tissue, prostatic stroma, and bilateral seminal vesicles (Figure 1). Perineural and lymphovascular invasion were also present. A positive margin involving the soft tissue was observed. One lymph node with extranodal extension was observed among 27 excised lymph nodes, staging the tumor as PT4aN1M0.

The patient was planned for adjuvant RT, but due to a long waiting list, he was given first-line adjuvant gemcitabine 1000 mg/m^2^ on days 1 and 8 and cisplatin 100 mg/m^2^ on day 1, in combination, as a three-weekly regimen per the current chemotherapy protocol. He was planned for two cycles of adjuvant chemotherapy but could receive only one cycle due to low hemoglobin and the unavailability of a donor. Laboratory findings are summarized in Table 1. He was managed conservatively with hematinic and then planned for adjuvant RT. There was a two-month interval between surgery and commencement of adjuvant RT due to treatment wait times.

**Table 1:** Laboratory findings with normal range Hb: hemoglobin; TLC: total leukocyte count; PLT: platelet count; RBS: random blood sugar; Sr. creat: serum creatinine; ALP: alkaline phosphatase

Test	Result	Units	Reference Values
Hb	7.5	g/dL	11.0-16.0
TLC	4900	/uL	4500-11000
PLT	2.04	10^9^/L	1.00-3.00
RBS	81	mg/dL	70-140
Sr. creat	0.88	mg/dL	0.6-1.1
ALP	221	U/L	43-138

**Figure 2:**
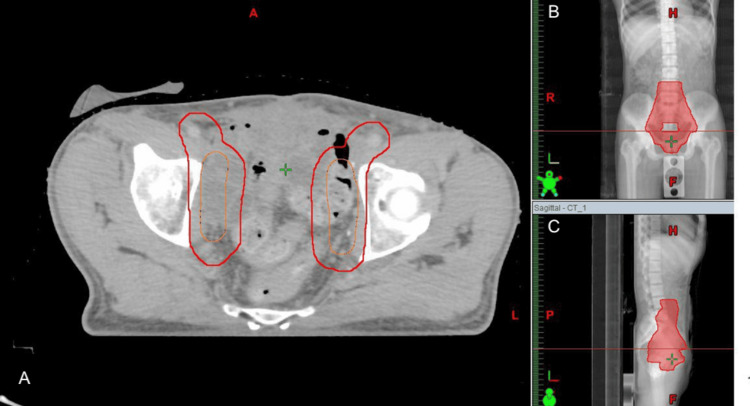
(A) Axial, (B) coronal, and (C) sagittal views of the pelvic nodal clinical target volume

**Figure 3:**
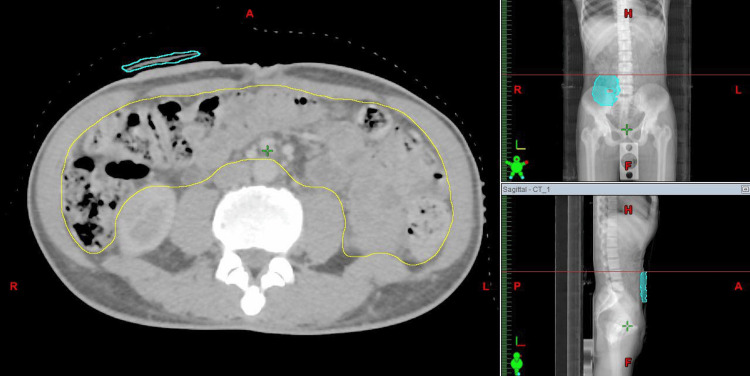
Representative axial computed tomography slice demonstrating the ostomy bag (blue) and bowel (yellow)

**Figure 4:**
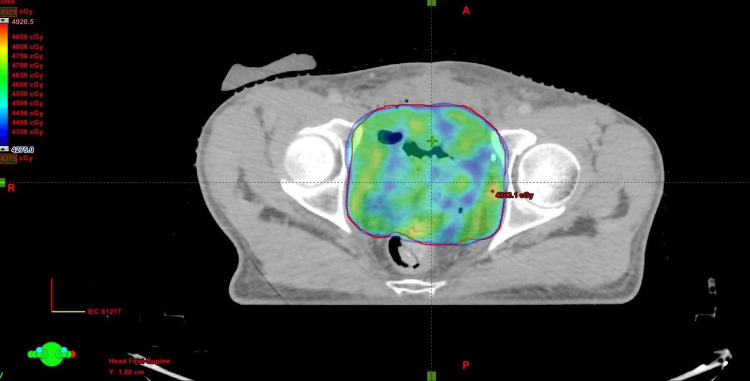
Volumetric modulated arc therapy (VMAT) treatment plan demonstrating dose distribution in an axial section

We used volumetric modulated arc therapy (VMAT) to plan radiation treatment, and a dose of 45 Gy in 25 fractions to the postoperative bed and regional pelvic lymph nodes was delivered (Figure 2). Organs at risk were contoured (Figure 3), and plan optimized and evaluated with proper dose constraints, and treatment was delivered. Dose distribution is shown in Figure 4. An electronic portal imaging device (EPID) was used to acquire daily 2D portal images. He was treated on a 6MV linear accelerator with weekly concurrent cisplatin at a dose of 40 mg/m^2^. During the course of RT, grade 1 skin, grade 2 hematological, and grade 2 lower gastrointestinal acute toxicities were observed, which were managed with supportive care. Only three cycles of concurrent cisplatin could be administered due to toxicity.

One month after completion of concurrent chemoradiotherapy (CTRT), a whole-body positron emission tomography (PET) scan demonstrated sclerotic lesions in the left iliac bone with increased fluorodeoxyglucose (FDG) uptake, raising suspicion for metastatic disease. Palliative RT was administered to the bone lesions at a total dose of 20 Gy in five fractions.

**Figure 5:**
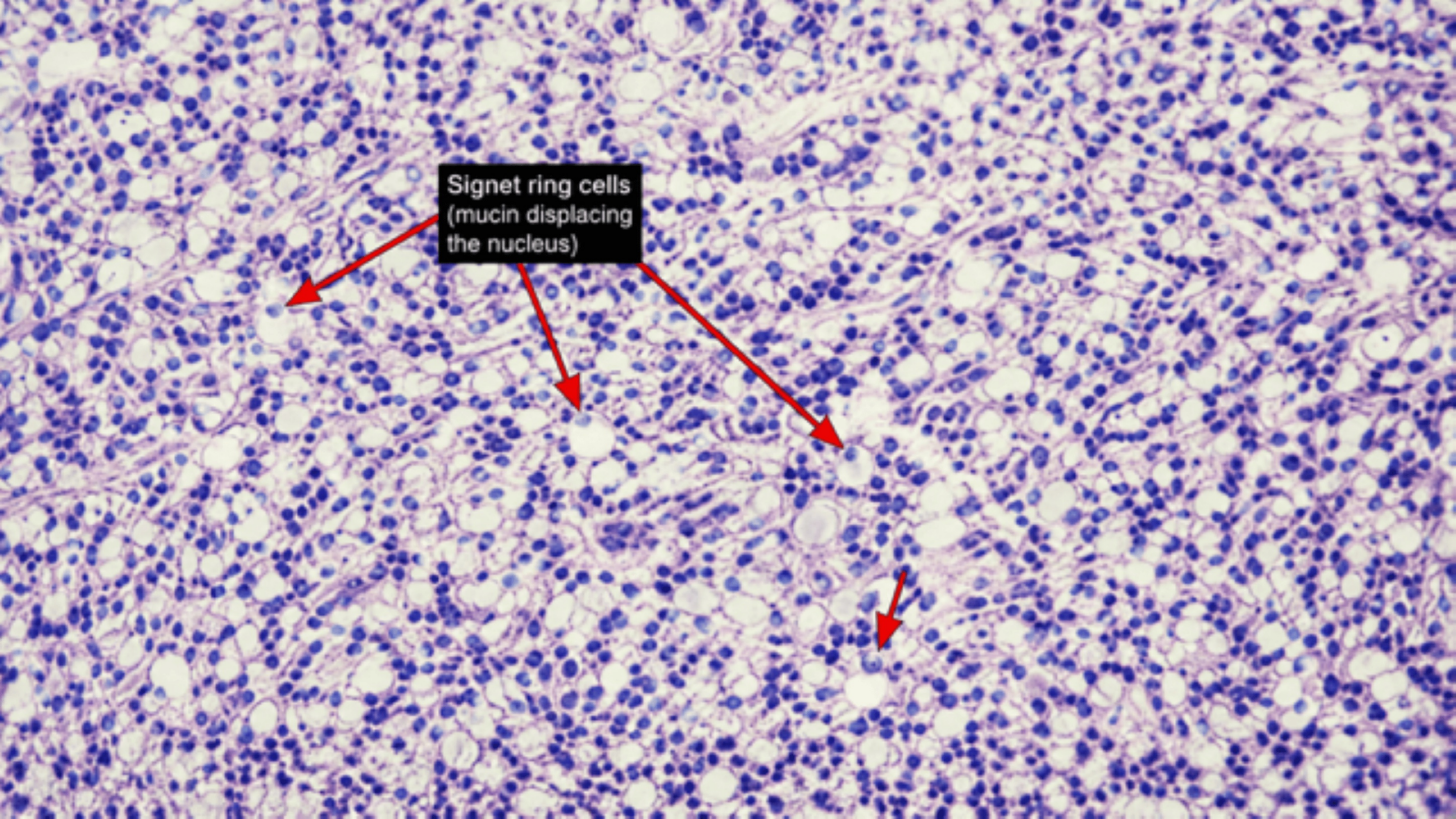
Hematoxylin and eosin stain showing mucin pools or lake with signet-ring morphology

He was then started on oral capecitabine 500 mg daily with intravenous zoledronate 4 mg once monthly. During the course of palliative treatment, nine months post chemoradiation, he developed 2 x 1 cm hard swellings on the glans penis. Biopsy from the growth over the glans penis and shaft of the penis revealed metastatic signet-ring cell carcinoma (SRCC) (Figure 5). A whole-body PET scan was performed again and revealed penile metastasis. In view of progressive disease, oral capecitabine was stopped and shifted to intravenous cisplatin.

**Figure 6:**
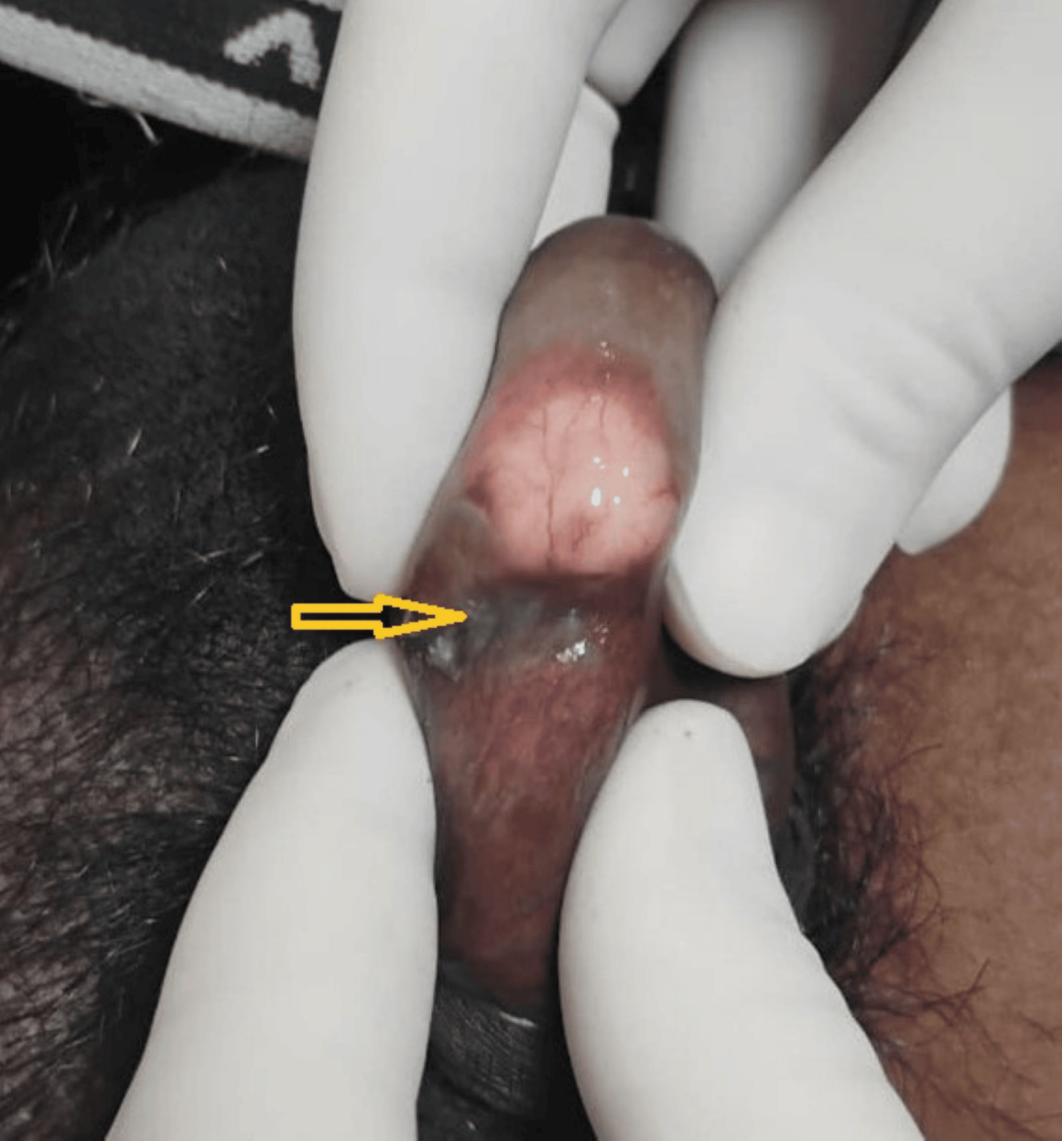
Penile metastatic lesion (arrow) involving the glans and shaft in a patient that developed during the course of treatment, which revealed signet-ring cell carcinoma on biopsy

Presently, he is alive and has received two cycles of cisplatin and four cycles of zoledronate, which were well tolerated, but his hemoglobin level decreased and is being managed with blood transfusion and hematinic. The penile lesion is shown in Figure 6.

## Discussion

PSRCC of the urinary bladder is a rare form of adenocarcinoma, accounting for only 0.12% to 0.6% of bladder cancers, with adenocarcinomas themselves comprising merely 0.5-2% of epithelial bladder malignancies [9]. Bladder SRCC typically presents in the sixth decade of life, predominantly affecting males, with higher incidences seen in males than in females [10]. The most common clinical symptom seen is hematuria, along with other symptoms like suprapubic pain, dysuria, and frequent urination [10]. At diagnosis, about one-fourth of the patients present with distant metastases [1].

The highly aggressive biologic behavior of SRCC often leads to frequent extravesical soft-tissue extension [9,11]. Histologically, SRCs are poorly differentiated, round cell tumors with intracytoplasmic mucin without extracellular mucin, resembling a mammary lobular carcinoma except for large cell size [12]. Diagnosing primary bladder SRC requires thorough clinicopathological correlation and, most importantly, exclusion of another primary tumor site due to its extremely low incidence [9]. A comprehensive clinical and radiological assessment is essential whenever primary bladder adenocarcinoma is suspected to differentiate it from secondary bladder involvement through direct extension or metastatic spread. Recommended workup includes PET/CT, pelvic MRI, upper endoscopy, and colonoscopy, along with mammography and gynecologic examination where appropriate. Evaluation should focus on ruling out primary malignancies arising from the gastrointestinal tract, including colon, appendix, stomach, as well as prostate, genital tract, and breast [9]. Reported imaging findings of bladder adenocarcinoma include diffuse or focal bladder wall thickening and adjacent fat stranding [13].

It has been suggested that radical cystectomy with ileal bladder conduit should be considered the preferred therapeutic approach for bladder SRCC [14]. Akamatsu et al. [1] reported that intravesical Bacillus Calmette-Guérin (BCG) therapy provides no benefit in SRCC of the bladder. Several studies have highlighted the inherent resistance of SRCC to conventional chemotherapy, resulting in inconsistent therapeutic outcomes [15,16]. In our case, the patient underwent radical cystectomy with an ileal conduit. Following surgery, the need for adjuvant therapy remains largely investigational, and published outcomes are often inconsistent.

Reddy et al. [9] recommended the addition of adjuvant RT and chemotherapy after surgery, noting improved local control and a reduced likelihood of recurrence. Encouraging outcomes with multimodal treatment have also been described in case reports. Hamakawa et al. [17] presented a patient with T3bN0M0 disease who underwent three cycles of postoperative cisplatin plus (tegafur, gimeracil, oteracil) and remained disease-free for 90 months. Likewise, Cobo-Dols et al. [18] reported a pT4aN0M0 case managed with cisplatin and gemcitabine that showed no recurrence at eight months. Ota et al. [19] also demonstrated benefit from intra-arterial methotrexate and cisplatin in combination with postoperative RT. Nonetheless, a standardized chemotherapy protocol for bladder SRCC has yet to be defined. Adjuvant chemotherapy may be considered for patients with good performance status who are at significant risk for distant metastasis [9].

Serum carcinoembryonic antigen (CEA) levels have also been reported in these cases. Rising postoperative serum CEA levels have been associated with disease progression and may serve as a useful marker for assessing tumor aggressiveness and follow-up monitoring [20]. In our case, serum CEA levels were not noted.

Adjuvant RT is generally considered when factors associated with increased recurrence risk are present, such as positive surgical margins or perivesical soft tissue involvement [9]. Since postoperative RT is commonly employed in gastrointestinal, gynecologic, and urologic cancers, including adenocarcinomas, to reduce pelvic recurrence, it is reasonable to anticipate a comparable benefit from adjuvant RT following cystectomy for bladder adenocarcinoma.

In patients with more locally advanced disease, adjuvant RT has demonstrated a statistically significant improvement in five-year disease-free survival (DFS) [9,21]. Although only a small proportion of patients in this cohort had SRCC, the greater tendency for locoregional recurrence in this subtype suggests that the benefit of adjuvant RT could be even more pronounced [9].

In a mixed-pathology cohort that included bladder adenocarcinoma, Zaghloul et al. [22] demonstrated that postoperative RT improved both DFS and local control across tumor types, stages, and histological grades in patients with P3a, P3b, or P4a disease. Patients either received hyperfractionated RT (1.25 Gy delivered thrice daily to a total of 37.5 Gy over 12 days), conventionally fractionated RT (50 Gy over five weeks), or no post-op adjuvant treatment [9]. The five-year local control rates were 50% with surgery alone, compared with 87% with hyperfractionation and 93% with conventional fractionation [22]. Even in transitional cell carcinoma, which generally behaves less aggressively than SRC, pelvic recurrence following cystectomy remains a significant problem, with rates approaching 30% in some studies [23]. Based on such evidence, Murthy and Zaghloul [24] have recently advocated revisiting the role of adjuvant RT after cystectomy, citing its potential not only to reduce the risk of local relapse but also to improve long-term outcomes. In our case, we delivered a conventional dose of 45Gy in 25 fractions concurrently with weekly cisplatin.

Standard chemotherapy regimens used for advanced urothelial carcinoma are generally considered not very effective in SRCC of the bladder. Authors reported that combination chemotherapy regimens such as epirubicin, cisplatin, and capecitabine (ECX), cisplatin and capecitabine (CX), epirubicin, oxaliplatin, and capecitabine (EOX), and irinotecan and cisplatin (IC) appear to offer superior efficacy compared with earlier standard regimens [25]. Kazaz et al. [26], in a case of gastric cancer with urinary bladder cancer of SRC histology, used four cycles of 5-fluorouracil (5-FU) and calcium folinate and six cycles of capecitabine and oxaliplatin in an adjuvant setting. Subsequently, three cycles of irinotecan and capecitabine, followed by three cycles of irinotecan, capecitabine, and oxaliplatin, were administered to the patient. The patient was disease-free at six months post-chemotherapy.

Case reports of SRCC originating from various primary sites, as well as bladder adenocarcinomas, commonly show that when responses to treatment occur, they are most often seen with 5-FU-based chemotherapy regimens. For instance, Michels et al. [27] documented a patient with metastatic bladder SRC who, after failing standard gemcitabine/carboplatin therapy, achieved meaningful progression-free survival with capecitabine, an oral 5-FU prodrug. Additionally, Hinduja et al. reported a case of stage IV bladder SRC treated successfully with FOLFOX (5-FU combined with oxaliplatin), resulting in a complete and sustained response [28].

In a reported series of 12 bladder SRCC cases managed with adjuvant or palliative chemotherapy, regimens included carboplatin, cisplatin, gemcitabine, capecitabine, and 5-FU [29]. Our patient had metastasis in the bone and penis one month and nine months post chemoradiation, respectively. We started him on oral capecitabine followed by cisplatin. He has received two cycles of cisplatin and four cycles of intravenous zoledronic acid and is alive.

## Conclusions

PSRCCs of the bladder are rare and have a poor prognosis. A comprehensive clinical and radiological evaluation is essential in suspected PSRCC cases to accurately stage the disease and exclude primary or metastatic bladder involvement originating from another malignancy. Optimal treatment has not yet been standardized due to its rarity, the paucity of case reports, and the absence of randomized trials. Addition of RT in tumors with positive margins and pT4a disease post-surgery and chemotherapy has been shown to improve locoregional control with a reduction in the risk of pelvic recurrences. Further work and studies are required to determine the appropriate management of these rare tumors.
